# Propofol-fentanyl versus propofol-dexmedetomidine in outpatient procedures sedation: a triple-blind, randomized controlled clinical trial

**DOI:** 10.1016/j.bjane.2025.844636

**Published:** 2025-05-03

**Authors:** Nicole Morem Pilau Moritz, Getúlio Rodrigues de Oliveira Filho, José Eduardo Moritz, Jefferson Luiz Traebert

**Affiliations:** a Hospital Universitário da Universidade Federal de Santa Catarina, Departamento de Anestesia, Florianópolis, SC, Brazil; b Universidade do Sul de Santa Catarina, Programa de Pós-Graduação em Ciências da Saúde, Palhoça, SC, Brazil; c Ultralitho Centro Médico, Florianópolis, SC, Brazil

**Keywords:** Anesthesia, Dexmedetomidine, Fentanyl, Outpatient surgical procedures, Patient satisfaction, Propofol

## Abstract

**Introduction:**

The choice of anesthetic agents plays a crucial role in procedural success. This study aimed to compare the effects of propofol-fentanyl and propofol-dexmedetomidine combinations, focusing on patient and surgeon perspectives in outpatient procedures.

**Methods:**

A randomized, controlled, triple-blind clinical trial including 128 adult patients undergoing elective outpatient surgical procedures with sedation and local anesthesia. Patients were randomized to receive either propofol-fentanyl (PF, n = 64) or propofol-dexmedetomidine (PDex, n = 64). Primary outcomes were patient satisfaction, assessed using the ISAS-Br score, and the adequacy of sedation, evaluated by the surgeon and measured by a Numerical Rating Scale (NRS) for movement. Respiratory and hemodynamic changes, as well as awakening from anesthesia, adverse events during recovery, and time to hospital discharge were compared.

**Results:**

No difference between patient satisfaction scores (median ISAS-Br [IQR]: PF 2.64 [2.45‒3.00] vs. PDex 3.00 [2.45‒3.00], p = 0.252). The PF group had a significantly lower movement score (median NRS [IQR]: 0.5 [0.00‒2.25] vs. 2.0 [0.00‒5.00], p = 0.006). The incidence of intraoperative events related to respiration and hemodynamics, as postoperative pain and postoperative nausea/vomiting were similar. A higher proportion of patients sedated with PF awoke in the operating room (75% vs. 35.9%, p < 0.001), and 98.4% of the PF group vs. 92.2% of the PDex group were ready for hospital discharge in less than thirty minutes, p = 0.208.

**Conclusion:**

Single doses of fentanyl or dexmedetomidine combined with propofol resulted in equivalent patient satisfaction, safety, and discharge times. The propofol-fentanyl combination demonstrated superior sedation adequacy from the surgeon’s perspective and facilitated a faster emergence from anesthesia.

## Introduction

Outpatient surgery has become an effective and cost-efficient alternative for many surgical procedures, offering advantages such as shorter recovery times, reduced risk of hospital-acquired infections, and higher patient satisfaction.^1^^,^^2^ The choice of anesthetic agents plays a crucial role in procedural success, ensuring both patient safety and surgeon satisfaction.^3^^,^^4^ Given the variety of available agents, understanding their benefits and risks is essential for optimizing sedation while minimizing adverse effects.^2^^,^^5^ Evidence suggests that no single agent is ideal for all aspects of anesthesia, necessitating multimodal approaches to enhance effectiveness and safety.^6^

Propofol is widely used in outpatient anesthesia due to its rapid onset, short recovery time, and antiemetic properties.^7^ However, its lack of analgesia requires combination with other agents, such as fentanyl, a potent opioid with fast onset and short duration.^7^, ^8^, ^9^, ^10^ While effective, fentanyl is associated with respiratory depression, chest rigidity, and nausea.^11^ Dexmedetomidine, a selective alpha-2 adrenergic agonist, has gained interest for its sedative, anxiolytic, and opioid-sparing effects, with minimal respiratory depression.^12^

Studies have reported high patient and surgeon satisfaction with dexmedetomidine sedation. However, when used alone, it may not achieve the desired depth and consistency of sedation, making combination with other agents necessary.^13^, ^14^, ^15^, ^16^, ^17^ While most research has focused on dexmedetomidine in endoscopic procedures, data on its combination with propofol in outpatient surgeries remain limited. Given dexmedetomidine’s ventilation-sparing properties compared to fentanyl’s respiratory depressant effects, we hypothesized that a single dose of dexmedetomidine with propofol might provide more consistent sedation than propofol-fentanyl.

This study aimed to compare the effects of propofol-dexmedetomidine and propofol-fentanyl combinations on sedation quality, evaluating outcomes from both patient and surgeon perspectives in outpatient procedures.

## Methods

This randomized, controlled, parallel-group, triple-blind superiority trial was conducted at Professor Polydoro Ernani de São Thiago University Hospital, Florianópolis, Brazil, a public tertiary health care center, between November 2022 and January 2024. The study was approved by the Research Ethics Committee of the Federal University of Santa Catarina (CAAE: 60823722.1.0000.0121) and registered in the Brazilian Clinical Trials Registry (ReBEC: RBR-4m7cpb5). All participants provided written informed consent.

Patients aged 18–65 years, classified as American Society of Anesthesiologists (ASA) physical status I–III, scheduled for elective outpatient surgical procedures lasting at least 15 minutes under local anesthesia and sedation, were included. Only minimally invasive surgeries that could be performed exclusively under local anesthesia were included to minimize heterogeneity in procedural characteristics and pain stimuli. Exclusion criteria comprised cognitive, mental, or neurological disorders, allergy/hypersensitivity to study medications, pregnancy, alcohol or illicit drug abuse, renal or hepatic dysfunction, heart rate < 50 bpm, systolic blood pressure < 90 mmHg, pacemaker use, and conversion to general anesthesia.

Before recruitment, patients were randomized 1:1 to receive either Propofol-Fentanyl (PF) or Propofol-Dexmedetomidine (PDex) using computer-generated block randomization (block size = 4) by Research Randomizer (www.randomizer.org). Group assignments were concealed in sealed, opaque, sequentially numbered envelopes, stored in the hospital pharmacy. Patients were recruited from the surgical center, ambulatory surgery unit, and cath-lab unit based on eligibility criteria. The principal investigator personally approached potential participants, obtained informed consent, and assigned a sequential number, which corresponded to the envelope concealing predefined group allocation. An anesthesiology resident, uninvolved in other study phases, opened the envelope, prepared the study solution, and administered the blinded syringe labeled “study medication”. The principal investigator, responsible for anesthetic management, remained blinded to the treatment allocation. Surgeons, assistant anesthesiologist, data analysts, and the recovery room nurse were also blinded to group assignment.

Patients were monitored with continuous pulse oximetry for oxygen saturation, electrocardiography for Heart Rate (HR), and non-invasive blood pressure every five minutes. All remained in spontaneous breathing with supplemental oxygen (4 L.min^-1^ via nasal cannula). After establishing peripheral venous access, the study medication was infused over 10 minutes via an infusion pump. Dexmedetomidine was administered at 0.5 µg.kg^-^¹, and fentanyl at 1 µg.kg^-1^. Esketamine (0.2 mg.kg^-1^ bolus) was available as rescue sedation. To maintain blinding, study drugs were diluted in 0.9% saline to a total volume of 20 mL.

Following infusion, patients received intravenous lidocaine (1 mg.kg^-1^ without vasoconstrictor). Propofol infusion was initiated using target-controlled infusion, initially set at 0.5 µg.mL^-1^ at the effect site, following the Schnider pharmacokinetic model. The assistant anesthesiologist adjusted the infusion rate as needed to maintain Observer’s Assessment of Alertness/Sedation Scale (OAA/S) ≤ 4.^18^ Esketamine was titrated as needed, and ephedrine or atropine was administered for hemodynamic instability. Once adequate sedation was achieved (OAA/S ≤ 4), the surgeon administered local anesthesia (2% lidocaine, with or without vasoconstrictor). Tenoxicam, dipyrone, and ondansetron were administered if no contraindications were present.

Approximately 10 minutes before the end of the procedure, propofol infusion was discontinued. Total propofol and esketamine doses, procedure duration, and the surgeon-assessed movement score were recorded. Patients were then transferred to the Post-Anesthesia Care Unit (PACU), where they were monitored until full recovery. Adverse events such as pain, Postoperative Nausea and Vomiting (PONV), and hemodynamic instability were documented in the PACU, and their management was at the discretion of the assistant anesthesiologist. Before discharge, patients completed a satisfaction questionnaire, ensuring at least one hour had elapsed since anesthetic administration. The recovery room nurse, who was not involved in the study, administered the questionnaire to maintain blinding. Sociodemographic and clinical data were collected via interviews, self-administered questionnaires, and medical records.

Primary outcomes were patient satisfaction and the surgeon's assessment of sedation adequacy. Patient satisfaction was measured using The Iowa Satisfaction with Anesthesia Scale (ISAS),^19^ adapted for use in Brazil (ISAS-Br).^20^ This 11-item questionnaire employs a six-point Likert scale, generating a total satisfaction score ranging from -3 to +3. Sedation adequacy was assessed using a 10-point Numerical Rating Scale (NRS) for patient movement, where 0 = no movement and 10 = intense movement. Lower scores indicated better sedation adequacy and improved procedural conditions.

Secondary outcomes included the incidence of intraoperative respiratory and hemodynamic events, postoperative pain, PONV, and recovery time. Respiratory events were classified as airway obstruction or respiratory depression, defined as oxygen saturation < 90% or requiring chin lift, airway adjunct placement, or positive pressure ventilation. Hemodynamic instability was defined as a ≥ 30% variation in mean arterial pressure or HR from baseline, including hypotension, hypertension, bradycardia, or tachycardia. Each patient was counted only once per type of adverse event, regardless of recurrence. Recovery time was recorded as the interval from PACU arrival to readiness for discharge (OAA/S = 5) and categorized as ≤ 30 minutes or > 30 minutes.

### Statistical analysis

Summary statistics were reported as mean (Standard Deviation; SD) or median (interquartile range; IQR; 25^th^−75^th^ percentiles) for continuous variables and absolute and relative frequencies for categorical variables. The Shapiro-Wilk test assessed the distribution of continuous variables. Student’s *t*-test was applied for normally distributed data, while the Mann-Whitney *U* test was used for non-normal distributions. Pearson’s Chi-Square test or Fisher’s exact test analyzed differences in categorical data. A p-value < 0.05 was considered statistically significant.

Sample size calculations were based on both primary outcomes. A previous study comparing dexmedetomidine to placebo for sedation during local anesthesia reported an effect size of 0.5 standard deviations (Cohen’s *d* = 0.5).^13^ Using this effect size, a Type I error of 5%, a Type II error of 20% (power = 80%), and equal allocation (1:1 ratio), the required sample size was 64 participants per group (n = 128). Additionally, to detect a 20% difference in intraoperative movement scores (0–10 scale, where 0 = no movement and 10 = intense movement), we estimated an SD between 2.5 and 3.0.^21^ With a minimum detectable difference of 2 points, the required sample size per group ranged from 24 to 36 participants, depending on the assumed SD. Statistical analyses were performed using OpenEpi v3.01^22^ and Jamovi v2.3.9.^23^

## Results

**Figure 1:**
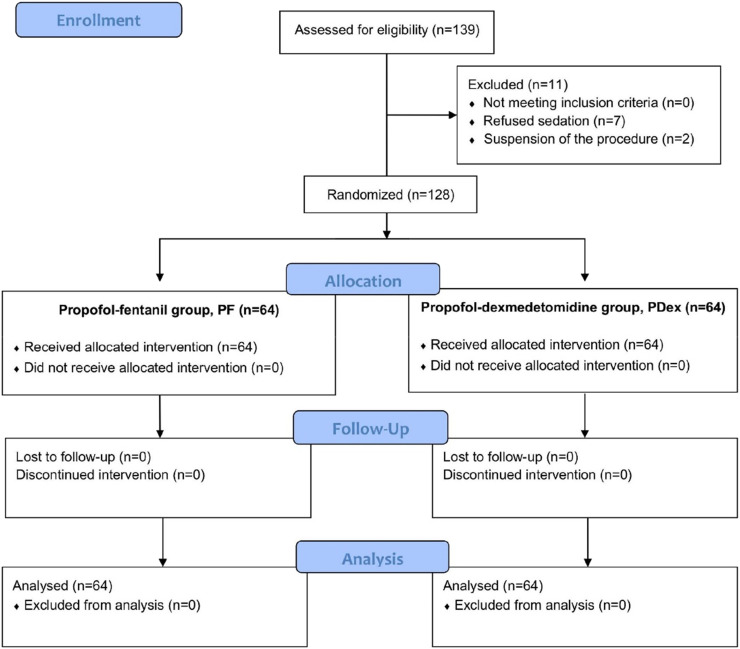
Flow Diagram according to CONSORT.

A total of 139 patients were recruited for the study, with 11 excluded before randomization. The remaining 128 patients were equally randomized into the PDex group (n = 64) and the PF group (n = 64), with all receiving the allocated intervention. No patients were excluded due to conversion to general anesthesia or protocol deviations (Fig. 1). Demographic and clinical characteristics were comparable between groups (Table 1). No data related to any of the outcomes was missing or incomplete.

**Table 1:** Demographic and clinical characteristics of patients.

Characteristic	PF group (n = 64)	PDex group (n = 64)
Age, median (IQR); years)	43.0 (33.0‒55.8)	43.5 (32.8‒53.3)
Sex, n (%)		
Male	19 (29.7)	19 (29.7)
Female	45 (70.3)	45 (70.3)
BMI, mean (SD); kg.m^-2^	27.2 (5.2)	26.3 (4.1)
Skin color, n (%)^a^		
White	47 (73.4)	43 (69.4)
Non-white	17 (26.6)	19 (30.6)
Education level, n (%)^a^		
4 to 8 years of schooling	17 (26.6)	13 (20.6)
9 or more years of schooling	47 (73.4)	50 (79.4)
ASA classification, n (%)		
I	15 (23.4)	16 (25.0)
II	46 (71.9)	42 (65.6)
III	3 (4.7)	6 (9.4)
Procedure duration, median (IQR); min	22.0 (15.0‒57.0)	26.5 (15.0‒51.0)
Propofol infusion duration, median (IQR); min^a^	27.0 (17.5‒53.5)	25.5 (17.0‒53.0)
Propofol dose, median (IQR); µ.kg^-1^.min^-1^^a^	115.9 (77.6‒178.4)	118.2 (70.2‒164.7)
Requirement for esketamine rescue, n (%)	6 (9.4)	14 (21.9)
Surgical specialties, n (%)		
Dermatology	1 (1.6)	1 (1.6)
General surgery	19 (29.6)	20 (31.2)
Gynecology	25 (39.0)	18 (28.1)
Ophthalmology	14 (21.9)	16 (25.0)
Plastic surgery	4 (6.3)	7 (10.9)
Head and neck surgery	‒	1 (1.6)
Urology	‒	1 (1.6)
Vascular surgery	1 (1.6)	‒

IQR, Interquartile Range (25^th^−75^th^ percentiles); SD, Standard Deviation; PF group, Propofol-Fentanyl group; PDex group, Propofol-Dexmedetomidine group.

a Skin color (n = 126; PDex n = 62), education level (n = 127; PDex n = 63), dose and duration of propofol infusion (n = 127; PF n = 63).

The ISAS-Br satisfaction score ranged from 1.36 to 3.00 in the PF group and 1.64 to 3.00 in the PDex group. The movement score ranged from 0 to 10 in the PF group and 0 to 9 in the PDex group (Table 2).

**Table 2:** Patient satisfaction and sedation adequacy from the surgeons' perspective.

Outcomes	PF Group (n = 64)	PDex Group (n = 64)	p-value	Effect size
ISAS-Br score, median (IQR)^a^	2.64 (2.45‒3.00)	3.00 (2.45‒3.00)	0.252	0.11
Movement score, median (IQR)^b^	0.50 (0.00‒2.25)	2.00 (0.00‒5.00)	0.006	0.27

A p-value < 0.05 was considered statistically significant. ISAS-Br, The Iowa Satisfaction with Anesthesia Scale; IQR, Interquartile Range (25^th^−75^th^ percentiles); PF group, Propofol-Fentanyl group; PDex group, Propofol-Dexmedetomidine group.

a Patient satisfaction measured by the ISAS-Br score (-3 to +3), where -3 indicates the lowest level of satisfaction and +3 the highest level of satisfaction. Mann-Whitney *U* test.

b Adequacy of sedation assessed by surgeons using a numerical rating scale (NRS; 0 to 10) for movement, where 0 indicates no movement and 10 indicates intense movement during the procedure. Mann-Whitney *U* test.

Adverse events related to respiration, hemodynamics, and recovery (including postoperative pain and PONV) were similar between groups (Table 3). Three patients in the PDex group experienced sialorrhea, with two requiring positive pressure ventilation for airway hyperreactivity and desaturation unresponsive to simple airway maneuvers. However, recovery was fast/quick. One patient required atropine, but not for bradycardia. Ephedrine was administered for hypotension in 5 (7.8%) patients in PDex and 1 (1.6%) in PF (p = 0.208).

**Table 3:** Incidence of adverse effects.

Outcomes	PF Group (n = 64)	PDex Group (n = 64)	p-value	RR (95% CI)
Respiratory events^a^	16 (25.0)	18 (28.1)	0.689	0.89 (0.5‒1.58)
Bradycardia^b^	‒	3 (4.7)	0.244	‒
Tachycardia^a^	12 (18.8)	5 (7.8)	0.068	2.40 (0.90‒6.42)
Hypotension^a^	21 (32.8)	29 (45.3)	0.147	0.72 (0.47‒1.13)
Hypertension^b^	4 (6.3)	‒	0.119	‒
Adverse events (PACU)				
Pain^b^	3 (4.7)	3 (4.7)	1.000	1.0 (0.21‒4.77)
PONV^b^	‒	1 (1.6)	1.000	‒

Data presented as count, n (%). A p-value < 0.05 was considered statistically significant. PF Group, Propofol-Fentanyl Group; PDex Group, Propofol-Dexmedetomidine Group; RR, Relative Risk; 95% CI, Confidence Interval; PACU, Post-Anesthesia Care Unit; PONV, Postoperative Nausea and Vomiting.

a Chi-Square test.

b Fisher's exact test.

Regarding esketamine use, 6 (9.4%) PF patients and 14 (21.9%) PDex patients required supplementation (p = 0.051). Among these, the median total dose was 15.5 mg (15.0‒22.0) in PF (range 14‒30 mg) and 14.0 mg (14.0‒15.0) in PDex (range 10‒30 mg), with no significant difference between groups (p = 0.090).

A significantly higher percentage of PF patients woke up in the operating room (48 [75.0%] vs. 23 [35.9%], p < 0.001). Recovery times were comparable, with 63 (98.4%) PF patients and 59 (92.2%) PDex patients ready for discharge within 30 minutes (p = 0.208).

## Discussion

This clinical trial demonstrated that single doses of fentanyl or dexmedetomidine, combined with propofol, resulted in similar patient satisfaction. However, Propofol-Fentanyl (PF) provided superior surgeon-rated sedation and facilitated a faster emergence from anesthesia. Both drug combinations had comparable respiratory and hemodynamic effects, with similar adverse event rates in the PACU and hospital discharge times in outpatient procedures with local anesthesia and sedation.

Patient satisfaction findings align with previous studies evaluating dexmedetomidine sedation, though different measurement tools were used.^16^^,^^17^^,^^24^ Some studies found higher satisfaction with dexmedetomidine, particularly when continuous infusion was used rather than a single dose, as in this study.^13^^,^^14^ One multicenter study compared dexmedetomidine infusion with placebo in various procedures, where all patients received fentanyl and midazolam as rescue medications.^13^ Another study compared dexmedetomidine to a propofol-alfentanil combination in ophthalmic surgery.^14^

The PF group had a significantly lower movement score, reflecting better sedation adequacy. The movement scale was selected for its objectivity and its importance in ensuring optimal procedural conditions. Various studies have assessed sedation adequacy using unvalidated Likert scales^16^, ^24^, ^25^ or numerical rating scales for procedural feasibility,^6^^,^^17^ which correlate inversely with movement scores. While some studies found no difference between dexmedetomidine and opioid-based sedation or propofol-dexmedetomidine *versus* ketamine-dexmedetomidine, others showed superior sedation quality with dexmedetomidine-propofol compared to propofol alone or propofol-remifentanil.^6^^,^^15^, ^16^, ^17^^,^^24^ However, inferior sedation quality was noted when dexmedetomidine was used alone *versus* propofol-fentanyl or its combination with ketamine *versus* ketamine-propofol.^25^^,^^26^

Respiratory events were similar across groups. These findings align with previous studies where dexmedetomidine was associated with fewer respiratory complications compared to opioid-based regimens.^6^^,^^16^^,^^17^^,^^27^ However, the use of propofol in both groups and the low fentanyl dose may explain why there were no significant differences. Two patients required positive pressure ventilation due to a decrease in oxygen saturation. Both events were attributed to airway hyperreactivity caused by sialorrhea, occurring exclusively during anesthesia emergence and therefore not linked to respiratory depression from anesthetic administration.

Although tachycardia and hypertension were more frequently observed in the PF group, while bradycardia and hypotension were more common in PDex, the differences were not statistically significant. Few cases required vasopressor intervention, and all of which were effectively managed. Notably, the dose-dependent bradycardia associated with dexmedetomidine treatment primarily results from reduced sympathetic tone, with additional contributions from the baroreceptor reflex and increased vagal activity.^12^ These findings support previous research demonstrating dexmedetomidine’s ability to maintain cardiovascular stability and its low incidence of severe hemodynamic events.^6^^,^^13^, ^14^, ^15^, ^16^, ^17^^,^^24^^,^^25^ However, it is important to consider that this study was not designed to detect differences in the incidence of adverse events between groups, and the sample size was not calculated for this purpose.

Adverse events in the PACU were low and comparable between groups, consistent with the literature.^6^^,^^13^^,^^17^^,^^24^^,^^25^ Both PF and PDex groups required similar rates of esketamine rescue, although a trend toward increased esketamine use in PDex may be attributed to higher movement scores in this group.

Patients in the PDex group were sleepier postoperatively, but this did not delay hospital discharge, likely due to the single dose of dexmedetomidine minimizing its sedative effects during recovery. In contrast, the faster emergence from anesthesia in the PF group has significant implications for ambulatory surgery efficiency. This outcome could potentially improve patient turnover, allowing surgical centers to accommodate more cases within the same timeframe. Additionally, it may optimize resource utilization, such as operating room time and staff availability, contributing to overall cost-effectiveness and operational efficiency in outpatient settings.

This study included various surgical procedures, enhancing the generalizability of findings to outpatient settings. However, since pain impacts patient satisfaction and can lead to movement during procedures, more invasive or painful surgeries could potentially affect the primary outcomes.^28^ To minimize variability, only minimally invasive surgeries feasible for local anesthesia were included, ensuring that sedation was administered solely for patient comfort rather than for intraoperative analgesia. This approach standardized nociceptive stimuli, reducing the need for additional sedatives or analgesics.

Furthermore, groups were balanced for key demographic factors that influence patient satisfaction, such as age and sex, as well as surgery duration, thereby strengthening the validity of comparisons.^29^ Nonetheless, differences in procedural types, sedation protocols, and sedation depth across different clinical trials should be considered when comparing results. This study underscores the importance of using validated assessment tools to evaluate sedation adequacy and patient satisfaction.^4^

The interpretation of secondary outcome results should be approached with caution due to the absence of dedicated sample size calculations. Additionally, ASA IV patients were excluded, as they typically require inpatient care. Future multicenter trials could help minimize selection bias.

One limitation was the use of single doses of fentanyl and dexmedetomidine, without restricting procedure duration. Since these drugs have different half-lives, prolonged surgical times could have differentially impacted the results. However, this potential bias was mitigated by the fact that procedure time, infusion time, and total propofol and esketamine doses were similar between groups.

Despite the study’s triple-blind design, pharmacodynamic differences between fentanyl and dexmedetomidine could have compromised blinding to some extent. Another important consideration is the choice of the Schnider pharmacokinetic model for propofol, despite the availability and widespread use of other models. The selection of a pharmacokinetic model depends on patient characteristics and the anesthesiologist’s preference. In this case, the decision was influenced by its association with dexmedetomidine, as a previous study demonstrated that dexmedetomidine reduced the target concentration required for induction and shortened induction time in the Schnider group compared to the Marsh model.^30^

While the groups were homogeneous regarding the quantity and duration of propofol administration, continuous sedation level monitoring was not conducted, limiting real-time control over sedation depth. Although the ISAS-Br is a validated tool, it has limitations in assessing deeply sedated patients.^20^ Future studies could incorporate Bispectral Index (BIS) monitoring to reduce variability in sedation levels and provide more objective evaluations of sedation depth and drug effects.

This study provides new insights into the sedation quality of fentanyl and dexmedetomidine when combined with propofol. The greater surgeon satisfaction and faster operating room discharge observed with PF suggest potential benefits in outpatient anesthesia workflows.

## Conclusion

In summary, our findings suggest that both fentanyl and dexmedetomidine, when combined with propofol, offer effective and safe sedation for outpatient procedures under local anesthesia. While fentanyl provided faster emergence and greater surgeon satisfaction, both regimens achieved high patient satisfaction and comparable safety profiles. These results support the use of either strategy depending on clinical priorities, and they encourage further research to optimize dosing and explore tailored sedation approaches in opioid-sparing contexts.

## Funding

This research did not receive any specific grant from funding agencies in the public, commercial, or not-for-profit sectors.

## CRediT authorship contribution statement

**Nicole Morem Pilau Moritz:** Conceptualization, Data curation, Formal analysis, Investigation, Methodology, Project administration, Resources, Supervision, Validation, Visualization, Writing – original draft, Writing – review & editing. **Getúlio Rodrigues de Oliveira Filho:** Data curation, Formal analysis, Methodology, Writing – original draft, Writing – review & editing. **José Eduardo Moritz:** Methodology, Writing – original draft, Writing – review & editing. **Jefferson Luiz Traebert:** Conceptualization, Formal analysis, Methodology, Project administration, Supervision, Writing – original draft, Writing – review & editing.

## Conflicts of interest

The authors declare no conflicts of interest.

## Appendix Supplementary materials

Image, application 1
