# Supplementary material for: Propofol-fentanyl versus propofol-dexmedetomidine in outpatient procedures sedation: a triple-blind, randomized controlled clinical trial
Source: Braz J Anesthesiol. 2025 May 3;75(4):844636. doi: 10.1016/j.bjane.2025.844636 (PMC12162015; doi:10.1016/j.bjane.2025.844636)
Supplement: Supplementary file 1 [file mmc1.docx]

**BJAN-D-24-00549_Supplementary Material**

**Supplementary Material Table S1** Procedure Type.

| **Procedure Type** | **PF group**  **(n = 64)** | **PDex group**  **(n = 64)** |
| --- | --- | --- |
| Lower limb angioplasty | 1 (1.6) | ‒ |
| Lymph node biopsy | 2 (3.1) | 1 (1.6) |
| High-frequency conization | 25 (39.1) | 18 (28.1) |
| Breast scar debridement | ‒ | 1 (1.6) |
| Arm dermolipectomy | ‒ | 1 (1.6) |
| Brow lift | 1 (1.6) | ‒ |
| Skin tumor excision | 3 (4.7) | 4 (6.3) |
| Breast implant cavity exploration | ‒ | 1 (1.6) |
| Unilateral gynecomastia surgery | ‒ | 1 (1.6) |
| Long-term venous catheter implantation | 14 (21.9) | 15 (23.4) |
| Bilateral otoplasty | 1 (1.6) | ‒ |
| Nasal ala reconstruction | 2 (3.1) | ‒ |
| Eyelid reconstruction | 14 (21.9) | 17 (26.6) |
| Scalp skin flap | ‒ | 1 (1.6) |
| Foreign body removal (right arm) | ‒ | 1 (1.6) |
| Long-term venous catheter removal | 1 (1.6) | 1 (1.6) |
| Abdominoplasty scar revision | ‒ | 1 (1.6) |
| Urethrotomy | ‒ | 1 (1.6) |

Data presented as count, n (%). PF Group, Propofol-Fentanyl Group; PDex Group, Propofol-Dexmedetomidine Group.
